# MicroRNA-15a-5p acts as a tumor suppressor in histiocytosis by mediating CXCL10-ERK-LIN28a-let-7 axis

**DOI:** 10.1038/s41375-021-01472-2

**Published:** 2021-11-16

**Authors:** Ran Weissman, Eli L. Diamond, Julien Haroche, Benjamin H. Durham, Fleur Cohen, Justin Buthorn, Zahir Amoura, Jean-François Emile, Roei D. Mazor, Noam Shomron, Omar I. Abdel-Wahab, Ofer Shpilberg, Oshrat Hershkovitz-Rokah

**Affiliations:** 1 Department of Molecular Biology, Faculty of Natural Sciences, Ariel University, Ariel, Israel; 2 Translational Research Lab, Assuta Medical Centers, Tel-Aviv, Israel; 3 Department of Neurology, Memorial Sloan Kettering Cancer Center, New York, NY USA; 4 Service de Médecine Interne, Hôpital Universitaire Pitié Salpêtrière - Charles Foix, Sorbonne Université, Faculté de Médecine, Paris, France; 5 Human Oncology and Pathogenesis Program, Memorial Sloan Kettering Cancer Center, New York, NY USA; 6 Department of Pathology, Memorial Sloan Kettering Cancer Center, New York, NY USA; 7 Research Unit EA4340, Versailles University, Paris-Saclay University, Boulogne, France; 8 Pathology Department, Ambroise Paré Hospital, Assistance Publique-Hôpitaux de Paris (AP-HP), Boulogne, France; 9 Institute of Hematology/Clinic of Histiocytic Neoplasms, Assuta Medical Centers, Tel-Aviv, Israel; 10 Faculty of Medicine and Edmond J. Safra Center for Bioinformatics, Tel Aviv University, Tel Aviv, Israel; 11 Adelson School of Medicine, Ariel University, Ariel, Israel

**Keywords:** Cell signalling, Haematological cancer

## Abstract

Erdheim–Chester disease (ECD) is characterized by excessive production and accumulation of histiocytes within multiple tissues and organs. ECD patients harbor recurrent mutations of genes associated with the RAS/RAF/MEK/ERK signaling pathway, particularly, the *BRAF*^*V600E*^ mutation. Following our previous finding that miR-15a-5p is the most prominently downregulated microRNA in ECD patients compared to healthy individuals, we elucidated its role in ECD pathogenesis. Bioinformatics analysis followed by a luciferase assay showed that chemokine ligand 10 (CXCL10) is a target gene regulated by miRNA-15a-5p. This was confirmed in 24/34 ECD patients that had low expression of miR-15a-5p concurrent with upregulated CXCL10. Overexpression of miR-15a-5p in cell lines harboring BRAF or RAS mutations (Ba/F3, KG-1a and OCI-AML3) resulted in CXCL10 downregulation, followed by *LIN28a* and p-ERK signaling downregulation and let-7 family upregulation. Overexpression of miR-15a-5p inhibited cell growth and induced apoptosis by decreasing Bcl-2 and Bcl-xl levels. Analysis of sequential samples from 7 ECD patients treated with MAPK inhibitors (vemurafenib/cobimetinib) for 4 months showed miR-15a-5p upregulation and CXCL10 downregulation. Our findings suggest that miR-15a-5p is a tumor suppressor in ECD through the CXCL10-ERK-LIN28a-let7 axis, highlighting another layer of post-transcriptional regulation in this disease. Upregulation of miR-15a-5p in ECD patients may have a potential therapeutic role.

## Introduction

Erdheim–Chester disease (ECD) is a rare histiocytic disorder with diverse clinical manifestations, ranging from indolent, localized presentations to a life-threatening, multi-system disease [1]. Histologically, ECD lesions consist of diffuse infiltration of foamy CD68+, CD1a− non–Langerhans cell histiocytes into bones and soft tissue (kidney, retroperitoneal space, skin, brain, and lung) [1]. Recurrent activating kinase mutations and fusions involving the ERK cascade and PI3K/AKT pathways and specifically, mutations in *BRAF* V600E and its downstream gene, *MAP2K1* [2, 3] have been discovered in a large proportion of ECD patients [1]. Treatments that block the pro-inflammatory pathway of IL-1 or the RAS/RAF/ MEK/ERK tumorigenic mutational network have shown efficacy in refractory ECD [4–6]; however, some of these treatments (e.g., the BRAF inhibitors, vemurafenib, and dabrafenib) demonstrated only partial efficacy and caused severe adverse effects [7, 8].

MicroRNAs (miRNAs) are short, non-coding RNAs that play a pivotal role in cancer initiation, progression, and treatment response via regulation of post-transcriptional gene expression (for a review, see Peng et al. [9]). We have recently showed that the miRNA profile of ECD patients differs from that of healthy controls [10]. Our analysis suggests that lower expression of certain miRNAs in ECD results in an upregulation of target genes that participate in cell survival signaling and inflammation. The most prominent and significant change detected between ECD and healthy controls was downregulation of miR-15a-5p, one of whose targets is the chemokine ligand 10 [CXCL10, also named interferon- inducible protein-10 (IP-10)] [11]. This chemokine is a member of the CXC chemokine family. CXCL10 specifically activates the CXCR3 receptor [12], which is predominantly expressed on activated T and B lymphocytes, natural killer (NK), dendritic cells, and macrophages [13]. CXCL10 is associated with a variety of human diseases including chronic inflammation, immune dysfunction, and tumor development and metastasis (for a review, see Liu et al. [14]).

The functions of miR-15a-5p have been investigated in hematological malignancies [15–17], but whether the dysregulation of this miRNA contributes to the pathogenesis and development of ECD has not yet been elucidated.

Here we evaluated the expression of CXCL10 in a large cohort of 34 ECD patients. Our results revealed that ECD patients have elevated levels of CXCL10, associated with downregulation of miR-15a-5p. We also demonstrated that increased expression of miR-15a-5p resulted in CXCL10 downregulation, followed by downregulation of the oncogene *LIN28a* and p-ERK signaling, leading to upregulation of the tumor suppressor let-7 family miRNAs. Furthermore, miR-15a-5p inhibited cell growth and induced apoptosis. Finally, analysis of sequential samples from 7 ECD patients treated with MAPK inhibitors (vemurafenib/cobimetinib) for 4 months resulted in upregulation of miR-15a-5p and downregulation of CXCL10.

These findings show that miR-15a-5p may function as a tumor suppressor in ECD patients through the CXCL10-ERK-LIN28a-let7 axis, highlighting an additional layer of post-transcriptional regulation in this disease. Upregulation of miR-15a-5p in ECD patients or in other myeloid malignancies with overexpression of the ERK cascade, may have a potential therapeutic role.

## Material and methods

### Samples

Plasma samples were collected from 10 healthy controls (HC) and 34 ECD patients. Seven samples were collected after 16 weeks of treatment. Samples were collected at Assuta Medical Center (Tel-Aviv, Israel), Memorial Sloan Kettering Cancer Center (MSK, New York, NY, USA), and Pitié-Salpêtrière Hospital (Paris, France) in accordance with local Institutional Review Board protocols. Blood samples were centrifuged at 1900 × *g* for 15 min and stored at −80 °C.

Excised lesions were fixed in 4% neutral-buffered formalin, embedded in paraffin, and processed by the routine procedures at each local department of pathology. Matched organ tissue biopsies of healthy donors were obtained by post-mortem autopsy.

### Cell lines

The human cell line KG-1a and OCI-AML-3 cells (kindly provided by Dr. Liran Shlush, The Weizmann institute of science, Rehovot, Israel) were cultured in Iscove’s Modified Dulbecco’s Medium (IMDM, Gibco, Thermo Fisher Scientific Inc., USA) supplemented with 20% fetal bovine serum (FBS, Biological industries, Israel). The cytokine-dependent murine pro-B Ba/F3 cells that stably express the MIGII- Empty Vector or MIGII- BRAF V600E (kindly provided by Dr Ben Durham, MSK, New York, NY, USA) were cultured in RPMI medium (Gibco, Thermo Fisher Scientific Inc., USA) supplemented with murine IL-3 (1 ng/ml, PeproTech, USA). Human embryonic kidney (HEK)−293 cells (kindly provided by Prof. Shai Izraeli, Schneider Children’s Medical Center, Petach Tikvah, Israel) were cultured in Dulbecco’s Modified Eagle’s medium (DMEM, Gibco, Thermo Fisher Scientific Inc., USA). Ba/F3 and HEK-293 media were supplemented with 10% FBS. All media were supplemented with 2 mM glutamine and 1% penicillin and streptomycin. Cells were cultured at 37^o^C in a humidified incubator with 5% CO_2._

### RNA extraction and quantitative RT-PCR

MiRNAs from human plasma samples, formalin fixed paraffin embedded (FFPE) tissue or cell lines were purified using the miRNeasy Serum/Plasma kit, miRNeasy FFPE kit and RNeasy Plus Kit (QIAGEN, Germany), respectively, according to the manufacturer’s protocol.

MiRNA was reverse-transcribed with miRNA specific stem-looped RT primers (Life Technologies, Thermo Fisher Scientific Inc.) and incubated for 30 min at 16 °C, 30 min at 42 °C, and 5 min at 85 °C. Detection of miRNAs was performed using the TaqMan® Small-RNA primer and probe sets (Applied Biosystems, Thermo Fisher Scientific Inc.). qRT-PCR was performed in duplicates by Step One Plus Real-Time PCR (Life Technologies; Thermo Fisher Scientific Inc.) under the following conditions: 95 °C for 20 s, followed by 40 cycles of 95 °C for 1 s, and 60 °C for 20 s. MiRNA expression was quantified relative to the expression of external synthetic cel-miR-39 or U6-snRNA (Applied Biosystems; Thermo Fisher Scientific Inc.), as internal controls.

For gene expression assay, RNA was isolated using the RNeasy Plus Kit (QIAGEN, Germany) and reverse transcribed using the high-capacity cDNA RT kit (Life Technologies; Thermo Fisher Scientific Inc.). qRT-PCR was performed in duplicate using Step One Plus Real-Time PCR (Life Technologies; Thermo Fisher Scientific Inc.) in a 10 µl reaction containing 30 ng cDNA, 5 µl TaqMan master mix (Life Technologies, Thermo Fisher Scientific Inc.), and 1 µl of FAM dye-labeled TaqMan probe of target gene or HPRT1 control primers (Life Technologies, Thermo Fisher Scientific Inc.). Reaction conditions for the gene expression assays were the same as for the miRNA qRT-PCR described above. Fold change was calculated using the ΔΔCt method.

For CXCL10 expression in FFPE tissue, we have run a pre-amplification reaction of cDNA using TaqMan Preamp Master Mix (Thermo Fisher Scientific Inc.) according to the manufacturer’s instructions. The diluted PreAmp product was used as a template for real-time PCR analysis as described above.

### Immunohistochemistry

Immunohistochemical analysis was performed on archival, FFPE histiocytosis tumor and non-histiocytosis control patient specimens. We used a Discovery Ultra instrument with a multimer/DAB detection system (Ventana Medical Systems Inc., Tucson, AZ, USA) with appropriate negative and positive controls using the following antibodies targeting human tissue: anti-CXCL10 (IP-10) (clone JA10-82; Host Species: Rabbit mAb; dilution: 1:200; Thermo Fisher Scientific, Waltham, MA, USA) and phospho-p44/42 MAPK (ERK1/2) (Thr202-Tyr204) (clone: D13.14.4E; Host Species: Rabbit mAb; dilution: 1:100; Cell Signaling Technologies, Danvers, MA, USA). Microscopic slides were evaluated using an OLYMPUS BX41 microscope (Olympus Scientific Solutions, Waltham, MA, USA), and images were acquired using an OLYMPUS DP72 camera (Olympus Scientific Solutions, Waltham, MA, USA).

### Enzyme-Linked Immunosorbent Assay (ELISA)

The concentrations of CXCL10 in plasma samples and conditioned medium from cells were determined by Human IP-10 (ab179194) or Mouse IP-10 (ab214563) ELISA kits (Abcam, United Kingdom). Human plasma or cell culture media (50 µl) were used as input for the assay. Standard curve samples and input samples were mixed with the appropriate antibody cocktail and were incubated with agitation for 1 h at room temperature. Next, wells were washed three times with wash buffer and TMB substrate was added and incubated for 15 minutes in the dark. Finally, stop solution was added to the plate and the absorbance (450 nm) was measured in an ELISA plate reader (Synergy HTX multi-mode reader, Winooski, Vermont, USA).

### Cell transfections with miRNA mimics/inhibitor

Cells were transfected for 48 h with 50 nM miR-15a-5p mimic or negative control (Applied Biosystems, Thermo Fisher Scientific Inc.) using lipofectamine 3000 reagent (Invitrogen, Thermo Fisher Scientific Inc.) according to the manufacturer’s protocol.

### Luciferase activity assay

The CXCL10 3’ UTR cloned into pEX-MT05 plasmid was purchased from GeneCopoeia, Inc., USA. HEK-293 cells were seeded 24 h before transfection. For the luciferase assay, 50 nM of negative control or miR-15a-5p mimic were co-transfected with the pEZX-MT05 vector containing the wildtype CXCL10 3’UTR (0.5 µg) using jetPRIME reagent (Polyplus transfection, Illkirch, France) according to the manufacturer’s protocol. Gaussian luciferase and alkaline phosphatase activities were measured by luminescence in conditioned medium, 48 h after transfection, using the secreted-pair dual luminescence kit according to the manufacturer’s instructions (GeneCopoeia, Inc., USA). Gaussian luciferase activity was normalized to alkaline phosphatase activity.

### Western blot

Cells were harvested using RIPA lysis buffer (Sigma Aldrich, USA) supplemented with protease inhibitor cocktail (Thermo Fisher Scientific Inc.). Equal amounts of protein were separated on a 10% SDS-PAGE gel (Bio-Rad, USA) and blotted onto nitrocellulose membranes. Membranes were blocked and incubated with the following primary antibodies: ERK (#4695), p-ERK [Thr202/Tyr204, (#9101)], Bcl-2 (#4223), Bcl-xl (#2764) and GAPDH (#97166) (Cell signaling technology, MA, USA). Secondary antibodies goat anti mouse and goat anti rabbit were purchased from Li-Cor Biosciences, USA.

### Cell proliferation assay

Growth inhibition by miR-15a-5p was measured by tetrazolium WST-1 assay (Roche, Basel, Switzerland). Cells were transfected with miR-15a-5p mimic or with a negative control. After 48 h of incubation, 10 µl of WST-1 reagent was added to the plate and incubated for 1 h at 37 °C, at which point the absorbance (450 nm) was measured in an ELISA plate reader (Synergy HTX multi-mode reader, Winooski, Vermont, USA).

### Apoptosis

Cells were assayed using the multifunctional Muse Annexin V and Dead Cell kit (Luminex / Millipore, Austin, Texas, USA). Cells were transfected with a miR-15a-5p mimic or negative control. After 48 h, cells were harvested, washed twice in PBS and 2 × 10^5^/100 µl were stained with 100 µl of Muse Annexin V and Dead Cell Reagent. Samples were incubated for 20 minutes at room temperature in the dark. Cells were analyzed by the Muse Cell-Analyzer (Millipore, Billerica, MA, USA) system and the percentage of apoptosis was determined using Guava software (Luminex/Millipore, USA, version 3.3).

### Cell-cycle analysis

Cells were assayed using the Muse Cell-Cycle kit (Luminex/Millipore, USA). Cells were transfected with miR-15a-5p or negative control. After 48 h, samples were washed twice in PBS and fixed in chilled 70% ethanol for 24 h. After ethanol removal, cells were suspended in 0.25 mL PBS per 5 × 10^5^ cells and warmed up to 37 °C. The cell pellet was resuspended in 200 µl of Muse Cell Cycle Reagent and incubated for 30 minutes at room temperature protected from light, then the cell suspension was transferred to a 1.5 ml microcentrifuge tube prior to analysis on Muse Cell Analyzer. Cell cycle was assayed by using Muse Cell Analyzer (Luminex/Millipore, USA). Percentage of cell-cycle stages was determined using Guava software (Luminex, USA, version 3.3). 

### Statistics

Data are presented as means ± SEM. Statistical comparison of means was performed by a two-tailed Student *t* test. Differences with *p* < 0.05 were considered statistically significant.

## Results

### MiR-15a-5p and CXCL10 expression in ECD patients

**Fig. 1:**
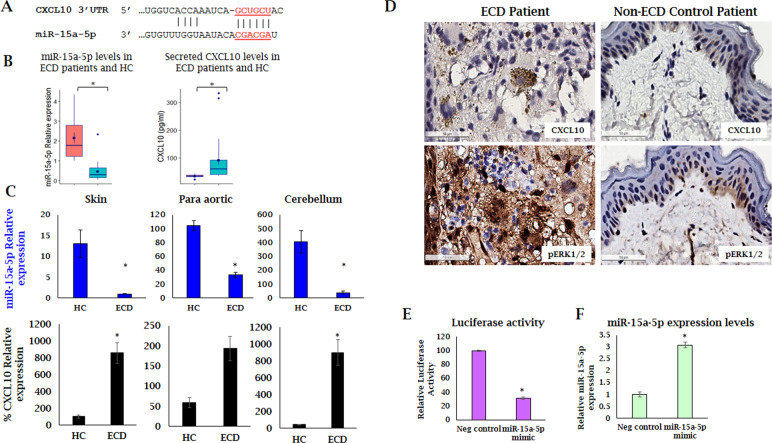
CXCL10 is highly expressed in ECD patients and is a target of miR-15a-5p. **A** Bioinformatics analysis (TargetScan) of the predicted miR-15a-5p binding site in the 3′UTR of the *CXCL10* gene. **B** MiR-15a-5p and secreted CXCL10 expression levels in plasma samples of ECD patients (green) and HC (red). ECD patients exhibit low levels of miR-15a-5p, as measured by qRT-PCR, normalized to cel-miR-39, and high levels of CXCL10 as measured by ELISA assay. **C** MiR-15a-5p and CXCL10 expression levels in ECD tissue samples and matched tissues from HCs. ECD patients exhibit low levels of miR-15a-5p (top), normalized to cel-miR-39, and high levels of CXCL10 (bottom), normalized to HPRT1, as measured by qRT-PCR. The histograms present the relative expression of miR-15a-5p and CXCL10 from at least 3 experiments. **D** Representative immunohistochemical images of skin from an ECD patient (left) showing overexpression of CXCL10 (top) and phospho-ERK1/2 (bottom) in neoplastic histiocytes compared to skin from a non-histiocytosis control patient (right) (anti-CXCL10 (IP-10) (top) and anti-phospho-ERK1/2 (bottom); 400× magnification; scale bars = 50 um). **E** HEK-293 cells were co transfected with miR-15a-5p mimic and reporter plasmid containing the wild-type 3’UTR of the CXCL10 gene. miR-15a-5p reduced luciferase activity by 70%. Data represent means of at least 3 experiments performed in triplicate and normalized to secreted alkaline phosphatase (SEAP). **F** The expression of miR-15a-5p in HEK-293 post transfection as measured by qRT-PCR. The histogram presents the relative expression of miR-15a-5p as normalized to U6 snRNA from at least 3 experiments. *p < 0.05. HC Healthy control, ECD Erdheim–Chester disease.

We have recently reported that miR-15a-5p is downregulated in plasma samples from ECD patients compared to healthy controls [10]. Patients’ characteristics are described in the Supplementary Table. Applying miRNA target scanning (using TargetScan [18]), we identified a potential binding site for miR-15a-5p in CXCL10 (Fig. 1A). We therefore examined the levels of CXCL10 and miR-15a-5p in 34 plasma samples obtained from ECD patients and from 10 HCs, by ELISA and qRT-PCR respectively. In accordance with our previous publication, we identified downregulation of miR-15a-5p in ECD compared to HC (Fig. 1B). In contrast, CXCL10 levels were upregulated in 24/34 ECD patients (70%) compared with HC (Fig. 1B). We further evaluated miR-15a-5p and CXCL10 by qRT-PCR and by immunohistochemistry in tissue biopsies of four ECD lesions (two skin biopsies, one para aortic and one cerebellum tissue biopsies as compared to matched healthy tissues from post-mortem autopsy). As in the plasma samples, miR-15a-5p was downregulated in ECD tissues and CXCL10 levels were upregulated compared with HC (Fig. 1C, D); strengthening our hypothesis that miR-15a-5p can potentially regulate CXCL10 expression.

### CXCL10 is a direct target of miR-15a-5p

To verify that CXCL10 is a bona fide target of miR-15a-5p, the 3′UTR of the human CXCL10 gene was cloned into the pEZX-MT05 reporter plasmid and was used in a dual luciferase reporter assay. This assay revealed that miR-15a-5p reduced luciferase activity by 70% (*p* value < 0.0001, Fig. 1E). MiR-15a-5p transfection efficiency, as measured by Taqman miRNA qRT-PCR, was found to be higher as compared to cells transfected with the scrambled negative control mimic (Fig. 1F). These results indicate that miR-15a-5p directly targets the 3’ UTR of the CXCL10 gene.

### Overexpression of miR-15a-5p downregulates CXCL10 levels

Several lines of evidence suggest that ECD originates from a myeloid progenitor [19–24]. We therefore investigated miR-15a-5p levels in the human myeloid cell lines KG-1a and OCI-AML3, which over express the MAPK-ERK pathway due to RAS mutations. In addition, those cells have been previously shown to express low levels of miR-15a-5p [25]. We also used the cytokine-dependent murine lymphoid pro-B Ba/F3 line that stably expresses the MIGII-BRAFV600E vector. Supplementary Fig. 1 shows the high levels of phosphorylated ERK (p-ERK) expressed in Ba/F3^*V600E*^, KG-1a, and OCI-AML3 cells compared to the parental Ba/F3 cells.

**Fig. 2:**
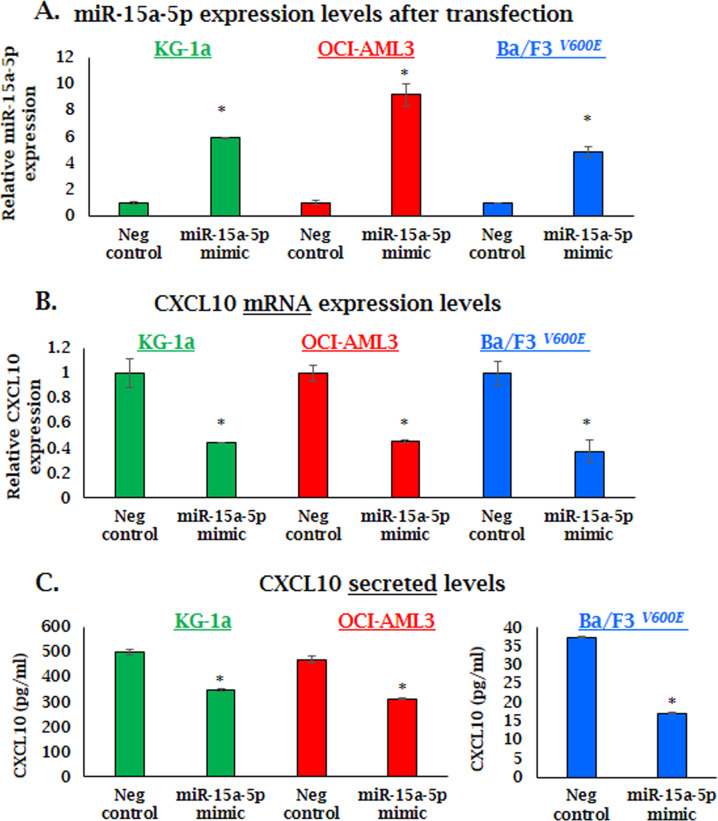
CXCL10 secreted and mRNA levels after miR-15a-5p mimic transfection. **A** KG-1a (green), OCI-AML3 (red), and Ba/F3 cells overexpressing the *BRAF*^*V600E*^ construct (blue), were transfected with a miR-15a-5p mimic (50 nM) or with a negative control for 48 h. The bars represent the expression of miR-15a-5p post-transfection as measured by qRT-PCR, normalized to snRNA U6 + SEM. **B** CXCL10 mRNA expression levels and (**C**) secreted expression levels were downregulated after transfection with a miR-15a-5p mimic in KG-1a, OCI-AML3, and Ba/F3^*V600E*^ cells. *p < 0.05.

Restoring miR-15a-5p levels by transfection with a miR-15a-5p mimic, upregulated miR-15a-5p in all 3 cell lines (Fig. 2A) followed by downregulation of *CXCL10* mRNA levels as measured by qRT-PCR (Fig. 2B) and downregulation of secreted CXCL10 levels as measured by ELISA (Fig. 2C).

### Low expression of CXCL10 results in downregulation of p-ERK signaling and target genes

**Fig. 3:**
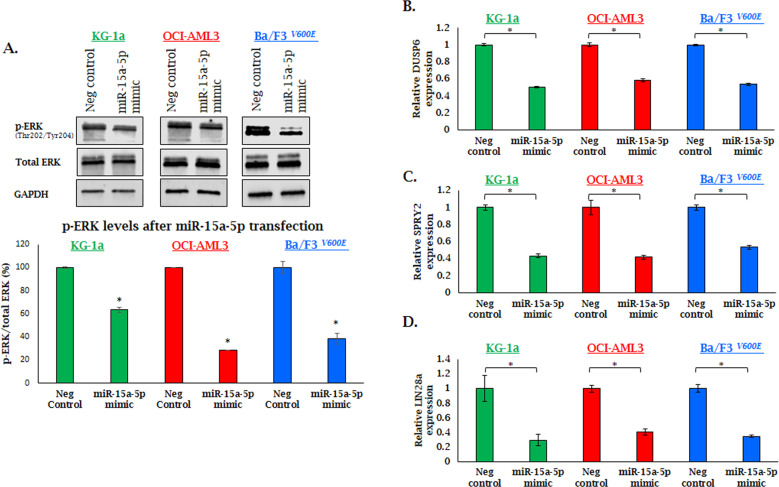
Protein levels of p-ERK and ERK-cascade target genes after miR-15a-5p transfection. KG-1a, OCI-AML3, and Ba/F3^*V600E*^ cells were transfected with a miR-15a-5p mimic (50 nM) or a negative control for 48 h. **A** p-ERK protein expression levels were downregulated after transfection with a miR-15a-5p mimic in all cell lines. Protein samples were resolved by SDS-PAGE. Antibodies to p-ERK, ERK, and GAPDH (loading control) were used to assay the levels of protein, which were then quantified by the Licor imaging system (bottom graph). The graph presents values from at least 3 experiments. **B** The expression of DUSP6, **C** SPRY2, and **D** LIN28a in KG-1a (green), OCI-AML3 (red) and Ba/F3^*V600E*^ (blue) cells post transfection as measured by qRT-PCR from at least 3 experiments. *p < 0.05.

Since high levels of CXCL10 were previously shown to activate the MAPK-ERK pathway in human glioma cells [26] and breast cancer cells [14, 27] we examined whether downregulation of this chemokine, following miR-15a-5p overexpression, affects the MAPK-ERK cascade in myeloid and lymphoid cells. As expected, expression of p-ERK was downregulated in all cell lines overexpressing miR-15a-5p (Fig. 3A). Next, in order to determine whether the downregulation of p-ERK is biologically relevant, we examined the expression of three representative ERK target genes (*DUSP6, SPRY2 and LIN28a*). The expression of these genes requires ERK signaling and therefore reflects a biologically relevant output of the BRAF-MEK-ERK signaling pathway [28, 29]. The expression of *DUSP6, SPRY2* and *LIN28a* mRNA levels was greatly reduced in KG-1a, OCI-AML3 and Ba/F3^*V600E*^ cells overexpressing miR-15a-5p (Fig. 3B–D), indicating a true biological effect of p-ERK downregulation.

### Inhibition of p-ERK signaling by miR-15a-5p and its effect on *LIN28a* and let-7 family miRNAs

**Fig. 4:**
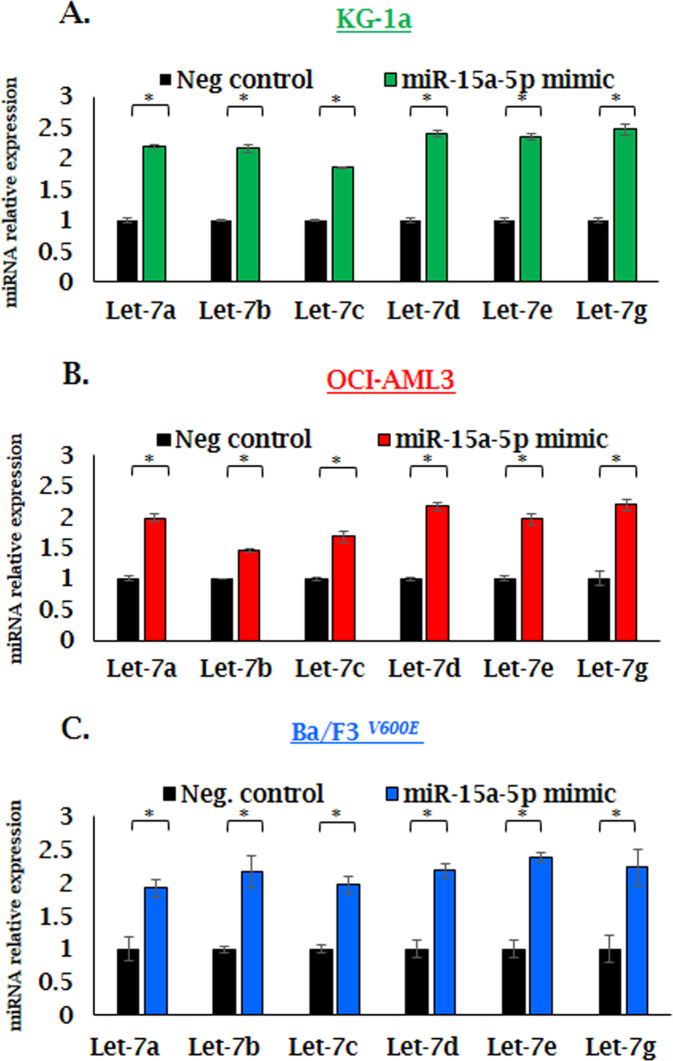
Let-7 family miRNA expression in the presence of miR-15a-5p. Upregulation of let-7a-5p, −7b-5p, −7c-5p, −7d-5p, −7e-5, and let-7g-5p after transfection of miR-15a-5p for 48 h. Expression of miRNA was measured by qRT-PCR, and normalized to U6 snRNA + SEM. Results are taken from at least 3 experiments. *p < 0.05.

Having established that the *LIN28a* oncogene is downregulated after miR-15a-5p transfection, we continued by analyzing the expression of the let-7 family miRNAs. We hypothesized that since miR-15a-5p expression leads to *CXCL10* downregulation, and subsequently in downregulation of p-ERK and *LIN28a*, we should observe upregulation of let-7 family miRNAs, which are regulated by the *LIN28a* oncogene. Fig. 4 shows that let-7 family miRNAs were indeed upregulated in all three cell lines after miR-15a-5p transfection.

### miR-15a-5p induces apoptosis and promotes growth arrest in cells overexpressing MAPK-ERK pathway

**Fig. 5:**
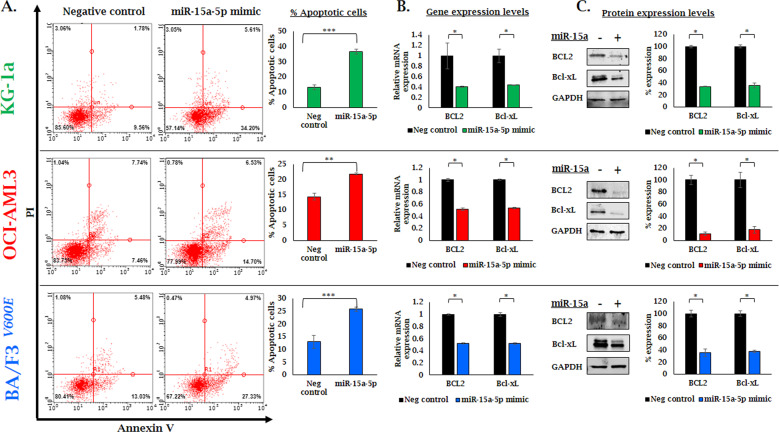
Over expression of miR-15a-5p induces apoptosis in-vitro. **A** miR-15a-5p induced apoptosis in KG-1a (green), OCI-AML3 (red), and Ba/F3^*V600E*^ (blue) cell lines. The cells were stained with Annexin V and propidium iodide (PI) and analyzed using the Muse cell-analyzer. The histograms on the right represent Annexin V-positive cells transfected with a miR-15a-5p mimic or a negative control. Values represent a mean of at least 3 experiments. **B** Reduced expression of mRNA and **C** protein levels of the pro-apoptotic Bcl-2 and Bcl-xl proteins. Protein samples were resolved by SDS-PAGE. Bcl-2, Bcl-xl and GAPDH (loading control) antibodies were used to assay protein levels, which were quantified by the Licor imaging system (right graph). Values of protein expression from at least 3 experiments. *p < 0.05, **p < 0.001, ***p < 0.0001.

Overexpression of miR-15a-5p resulted in a substantial increase in the rate of apoptosis in KG-1a, OCI-AML3 and Ba/F3^*V600E*^ cells as measured by flow cytometry after staining with Annexin V and PI (Fig. 5A). This was associated with downregulation of gene and protein expression levels of the pro-survival proteins Bcl-2 and Bcl-xl (Fig. 5B, C), which counteract apoptosis.

**Fig. 6:**
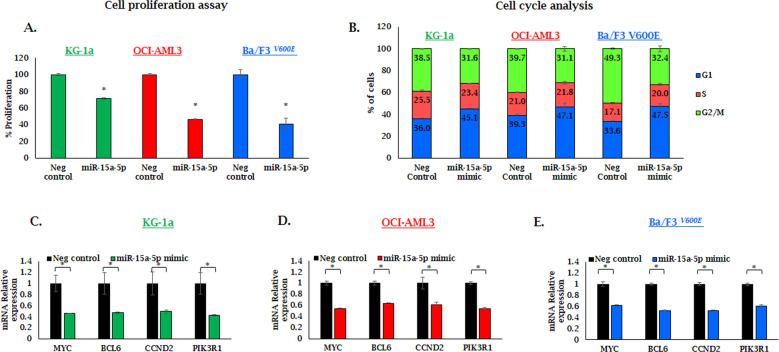
Over expression of miR-15a-5p reduced proliferation and oncogene expression. KG-1a (green), OCI-AML3 (red), and Ba/F3^*V600E*^ (blue) cells were transfected with a miR-15a-5p mimic or a negative control for 48 h. **A** Reduced proliferation of all cell lines overexpressing miR-15a-5p as measured by WST proliferation assay and (**B**) cell-cycle analysis. **C** Reduced oncogene expression leading to cell-cycle progression in (**C**) KG-1a, (**D**) OCI-AML3, and (**E**) Ba/F3^*V600E*^ cells as measured by qRT-PCR. The histograms present the relative expression of each gene from at least 3 experiments as normalized to HPRT1. *p < 0.05.

Overexpression of miR-15a-5p also decreased cell proliferation by 30–60% (Fig. 6A). Interestingly, this was reflected by a decrease in G2/M phase and an increase in G1 arrested cells that expressed miR-15a-5p as compared to controls (Fig. 6B). Oncogene transcription factors such as *MYC, BCL6, CCND2*, and *PIK3R1* were also downregulated following miR-15a-5p overexpression (Fig. 6C–E).

These results support our notion that miR-15a-5p increases apoptosis in these cells and alters the cell cycle distribution.

### MiR-15a-5p and CXCL10 expression in ECD patients after treatment

**Fig. 7:**
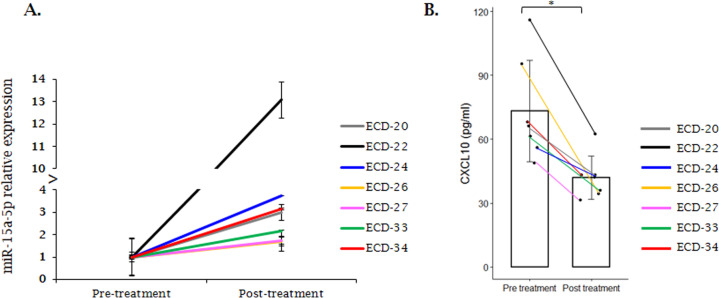
Upregulation of miR-15a and downregulation of CXCL10 after MEK inhibitor treatment. **A** miR-15a-5p was upregulated in seven patients after 16 weeks of targeted therapy as measured by qRT-PCR, in parallel to (**B**) downregulation of CXCL10 secreted levels as measured by ELISA. The histogram presents the relative expression of miR-15a-5p normalized to cel-miR-39 + SEM or CXCL10 + SEM from at least 3 experiments. *p < 0.05.

**Fig. 8:**
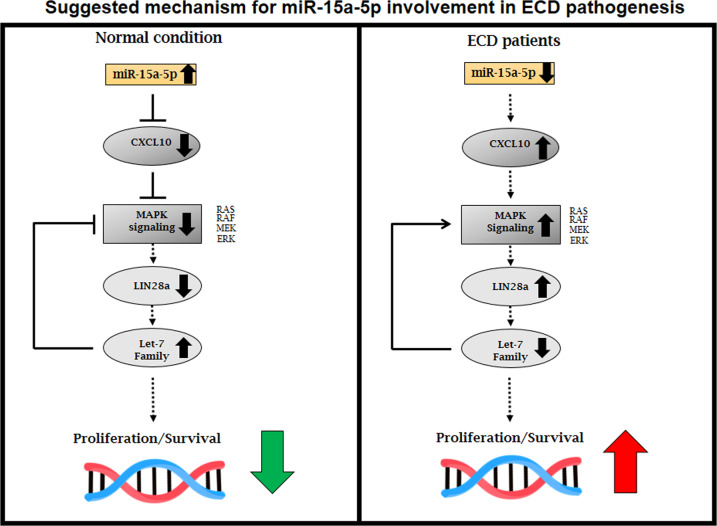
Suggested mechanism of the involvement of miR-15a-5p in the pathogenesis of ECD. Our Model suggests that downregulation of miR-15a-5p in ECD patients leads to  CXCL10 upregulation, which in turn increases MAPK-ERK (cascade) levels, thereby upregulating Lin28a expression. Consequently, let-7 family members are downregulated, enhancing the survival of aberrant cells.

As the next step, we examined whether the inhibition of the ERK-MAPK cascade seen in ECD patients treated with the MEK inhibitor (cobimetinib) has an effect on the levels of miR-15a-5p and CXCL10. To this end, we analyzed plasma samples from 7 patients, before and after, 16 weeks of treatment. We observed that miR-15a-5p was upregulated after treatment (Fig. 7A) and this was associated with a downregulation of CXCL10 (Fig. 7B) suggesting a potential feedback loop between miR-15a-5p-CXCL10 and the ERK cascade. Our suggested mechanism of the involvement of miR-15a-5p in the pathogenesis of ECD is shown in Fig. 8.

## Discussion

To date, the etiological mechanisms leading to ECD have not been fully understood. Previous studies reported that ECD histiocytes showed high expression levels of IL-6, which promotes activation and proliferation of macrophages [30]. Another study found high levels of IL-6 and IL-8 in mononuclear cells (MNC) extracted from ECD lesions [31]. IL-8 acts as chemoattractant for polymorphonuclear and monocyte cells, which are abundant in ECD lesions [32]. A different study reported higher levels of the pro-inflammatory cytokines, IFN-α, IL-12, IL-6, and MCP-1, in 15 untreated ECD patients compared to HC. These molecules are known to participate in Th1 cell differentiation and are necessary for systemic chemokine signaling [33]. High levels of CCL-18, which were measured in a cohort of 20 ECD patients, may explain the induction and progression of fibrosis observed in ECD lesions. CCL-18 has also been correlated with ECD severity [34]. In contrast, IL-7 and IL-4 were found to be downregulated in serum samples from ECD patients as compared to serum samples of HCs [33]. Interestingly, the same study revealed a signature of 5 cytokines (IL-1, IL-4, IL-7, IL-12 and IFN-α) that were elevated in untreated ECD patients with active disease compared to HC or individuals with other common inflammatory diseases such as rheumatoid arthritis, systemic sclerosis, or idiopathic inflammatory myopathies [33].

In this study, we examined the involvement of miR-15a-5p in the regulation of CXCL10, which is highly expressed in ECD patients. Since CXCL10 has multiple roles, including modulating the innate and adaptive immune response, cell growth regulation, and angiogenesis, we propose that the high expression of this chemokine in ECD may contribute to the neoplastic and inflammatory characteristics of the disease. A number of lines of evidence indicate ECD may originate from myeloid progenitors: (1) similar expression signatures and *BRAF* V600E mutations have been found in histiocytic neoplasms, blood monocytes, and hematopoietic stem cell/progenitors [19–21, 35, 36]. (2) histiocytosis-like lesions were generated by xenotransplantation of CD34^+^ cells from a patient with ECD [21], (3) ~10% of non-LCH patients have concurrent myeloid neoplasms [22]. Moreover, Ghobadi et al. [23] reported a patient with *BRAF* V600E-mutated ECD who developed acute monocytic leukemia (AML-M5). Whole exome sequencing confirmed that ECD and AML have multiple shared mutations arising from the same cell of origin, and (4) finally, a high frequency of clonal hematopoiesis among ECD patients was recently reported, reaffirming the risk for the development of additional myeloid neoplasms in ECD, and suggesting that many patients with ECD have defects originating in bone marrow precursors [24]. Here, we analyzed cell-lines harboring BRAF or RAS mutations (Ba/F3^*V600E*^, KG-1a and OCI- AML3), which results in enhanced MAPK signaling activation in a similar way to that observed in ECD patients. Experimental evidence demonstrated that correcting dysregulation of certain miRNAs by specific miRNA mimics or antagomirs can normalize the gene regulatory network and reverse the pathogenic phenotype seen in cancerous cells [37]. We therefore transfected KG-1a, OCI-AML3 and Ba/F3^*V600E*^ cells with miR-15a-5p mimic in order to investigate the effect on the CXCL10-ERK cascade. Overexpression of miR‐15a-5p suppressed cell proliferation through inhibition of the ERK-LIN28a axis, and induced apoptosis through inhibition of Bcl-2 family members. The conclusion that miR-15a-5p may act as a tumor suppressor is supported by a number of previous studies. The first evidence for miR-15a-5p involvement in cancer was described in chronic lymphocytic leukemia (CLL) where deletion of 13q14 was associated with downregulation of miR-15a in the majority of CLL patients [17]. In a different study, *Liu et al* showed that miR-15a over expression resulted in cell‐cycle arrest through the inhibition of CDK4 expression [38]. A significant inverse correlation between miR‐15a/16‐1 and WT1 expression levels was described in AML, suggesting that miR‐15a/16‐1 may inhibit leukemic cell proliferation through the downregulation of the WT1 oncogene [39]. Similarly, miR‐15a inhibits the proliferation of osteosarcoma cells, and this is thought to be through the downregulation of tumor necrosis factor α‐induced protein 1 (TNFAIP1) expression [40]. The miR-15/16 cluster was also found to target members of the mammalian target of rapamycin (mTOR) signaling pathway, including 4E‐BP1 and S6 kinase 1, which are important downstream effectors of mTOR signaling and control cellular growth and metabolism [41]. Overexpression of miR‐15/16 might lead to downregulation of mTOR pathway by targeting RPS6KB1. Interestingly, exogenous miR‐15/16 can control proliferation and induce apoptosis in a caspase‐dependent manner even in the absence of functional p53 [41]. Gianfreda et al. demonstrated mTOR pathway activation in ECD lesions and provided preliminary evidence of the efficacy of a sirolimus (mTOR inhibitor) and prednisone-based regimen in ECD patients [42, 43]. This suggests that the miR-15 cluster might be involved in the regulation of the mTOR pathway in ECD. Furthermore, Lovat et al [44] showed that knockout of both miR-15/16 loci induces AML in a mouse model. The complete deletion of both clusters promoted myeloproliferative disorders in the majority of the mice by the age of 5 months. Moreover, growth and therapeutic resistance in neuroblastoma was shown to be mediated through the miR-15a/16-1–ERK and Bcl-2/Cyclin D1 pathways [45] which supports our findings that miR-15a-5p may play a pivotal role in ECD development. Our results on the effect of miR-15a-5p on CXCL10 in ECD are supported by reports that miR-15a-5p also modulates neoplastic and immune/inflammatory characteristics in CML [11] and in the autoimmune disease myasthenia gravis [46]. In addition, CXCL10 was previously analyzed in a very small cohort of only three ECD patients by immunohistochemistry or in plasma samples [30, 47]. These studies reported over expression of CXCL10 in ECD patients, which is comparable to our results. A separate study that compared CXCL10 levels in the serum of 15 untreated ECD patients to those in 22 ECD patients treated with IFN-α, found no significant changes after treatment, or between ECD patients and healthy controls [33]. One possible explanation is the source of the samples, since in our study CXCL10 was measured in plasma and not in serum, suggesting that plasma may be a more appropriate media to examine CXCL10 levels compared to serum. Moreover, as the other study examined CXCL10 levels after INF-α treatment, our study examined CXCL10 levels after inhibition of the MAPK-ERK signaling by BRAF/MEK inhibitors, thus implying that CXCL10 expression levels are different between treatment types. Finally, the findings of upregulation of miR-15a-5p in all ECD patients after 16 weeks of targeted therapy with MEK inhibitors in parallel to downregulation of CXCL10 levels, suggest that miRNAs from peripheral blood may be useful as a simple monitoring tool for treatment response. However, sequential monitoring of miR-15a-5p and CXCL10 levels during the different treatment phases are required to determine whether these molecules may be used for clinical monitoring. Moreover, while our data shows that miR-15a-5p may have a central role in ECD, the study has several limitations: First, the cell-line models were not purified from ECD patients, however, we believe that the model we have chosen is the closest one to ECD because it is generated from myeloid progenitors, it harbor mutations in the MAPK signaling pathway, specifically in BRAF and RAS, which are common mutations found in ECD patients, and it expresses low levels of miR-15a-5p, as seen in ECD plasma and tissue samples. Nevertheless, we acknowledge that it would be important to further examine the involvement of miR-15a-5p in primary cultures purified from ECD patients. Second, as most experiments were performed on plasma samples, more tissue biopsies are needed to assess the exact role of miR-15a-5p and its influence on other inflammatory cytokines, and specifically on CXCR3 which is the receptor for CXCL10. Due to the scarcity of biological materials from ECD patients, we could not perform many experiments on the samples that we had from each patient. Lastly, as ECD is a systemic disease, it is important to acknowledge that other confounding factors or covariates were not analyzed.

Overall, our findings are consistent with miR-15a-5p’s role as a tumor suppressor that acts by downregulating CXCL10 and *LIN28a* expression through inhibition of the MAPK-ERK pathway. This study highlights additional layers of post-transcriptional regulation in ECD and suggests that upregulation of miR-15a-5p in ECD patients may have potential therapeutic utility in the management of this disease.

## Supplementary information

Supplementary Table 1

Supplementary Figure 1

Supplementary figure legend
