# Supplementary material for: MicroRNA-15a-5p acts as a tumor suppressor in histiocytosis by mediating CXCL10-ERK-LIN28a-let-7 axis
Source: Leukemia. 2021 Nov 16;36(4):1139–49. doi: 10.1038/s41375-021-01472-2 (PMC8979810; doi:10.1038/s41375-021-01472-2)
Supplement: Supplementary file 1 — Supplementary Table 1 [file 41375_2021_1472_MOESM1_ESM.docx]

**Supplementary Table 1. Patient's characteristics**

| **Name** | **Sex** | **Age** | **Sites** | **Mutation** |
| --- | --- | --- | --- | --- |
| ECD1 | Male | 75 | Bone, Peri-aortic Soft Tissue, Retroperitoneum | BRAF V600E |
| ECD2 | Male | 35 | Bone, Brain, Cavernous sinus, Peri-aortic Soft Tissue, Retroperitoneum, Right Atrium, Skull Base | BRAF V600E |
| ECD3 | Male | 50 | Bone, Brain, Peri-aortic Soft Tissue, Retroperitoneum, Right atrium | BRAF V600E |
| ECD4 | Male | 75 | Bone, Orbit, Retroperitoneum | BRAF V600E |
| ECD5 | Male | 58 | Bone, Orbit, Peri-aortic Soft Tissue, Retroperitoneum | BRAF V600E |
| ECD6 | Male | 55 | Bone, Brain, Heart, Retroperitoneum, Testes | BRAF V600E |
| ECD7 | Female | 56 | Bone, Brain, Retroperitoneum, Orbit | BRAF V600E |
| ECD8 | Female | 83 | Bone, Dura, Orbit, Retroperitoneum, Skin | BRAF V600E |
| ECD9 | Male | 53 | Bone, Retroperitoneum, Peri-aortic | BRAF V600E |
| ECD10 | Female | 52 | Bone, Brain | BRAF V600E |
| ECD11 | Male | 38 | Bone, Brain, Dura, Heart, Peri-aortic, Retroperitoneum, Skin, Skullbase | BRAF V600E |
| ECD12 | Male | 46 | Bone, Brain, Retroperitoneum | BRAF V600E |
| ECD13 | Male | 67 | Bone, Dura, Lymph Nodes, Peri-aortic, Retroperitoneum | NRAS Q61R |
| ECD14 | Female | 66 | Bone, Heart | KRAS G12S |
| ECD15 | Male | 69 | Bone, Retroperitoneum, Subcutaneous Soft Tissues | MAP2K2 Y134H |
| ECD16 | Male | 69 | Bone, Brain, Peri-aortic, Retroperitoneum | MAP2K1 C121S |
| ECD17 | Male | 18 | Bone, Brain, Dura | BRAF V600E |
| ECD18 | Female | 77 | Bone, Brain | BRAF V600E |
| ECD19 | Male | 51 | Bone, Larynx, Subcutaneous Soft Tissues | KRAS G12R / ARAF P216A |
| ECD20 | Male | 57 | Bone, Pituitary, Retroperitoneum, Spleen | MAP2K1 Q56P |
| ECD21 | Male | 54 | Bone, Brain, Heart, Retroperitoneum | BRAF V600E |
| ECD22 | Male | 47 | Bone, Brain, Heart, Pleura, Retroperitoneum, Spine | KRAS R149G |
| ECD23 | Female | 59 | Bone, Heart, Lymph Nodes, Pleura, Retroperitoneum, Skin | No mutation identified |
| ECD24 | Female | 66 | Bone, Heart, Peri-aortic | MAP2K1 P124Q |
| ECD25 | Female | 35 | Bone, Brain | BRAF N486_P490del |
| ECD26 | Male | 48 | Bone, Mucosa | Unknown |
| ECD27 | Female | 45 | Adrenal, Bone, Heart | BRAF V600E |
| ECD28 | Male | 58 | Brain, Sinus, Skin | BRAF V600E |
| ECD29 | Male | 63 | Kidney, Skin | Unknown |
| ECD30 | Male | 76 | Bone, Brain, Sinus | BRAF V600E |
| ECD31 | Female | 39 | Heart | BRAF V600E |
| *ECD32 | Female | 76 | Brain, Heart, Kidney, Lungs | BRAF V600E |
| ECD33 | Male | 71 | Kidney, Peri-aortic Soft Tissue, Retroperitoneum, | BRAF WT |
| ECD34 | Male | 34 | Bone, Brain, Kidney, Skin | BRAF V600E |

ECD, Erdheim-Chester Disease; M, Male; F, Female;

* Patient ECD32 has ECD + Chronic myelomonocytic leukemia (CMML).
