# Supplementary figures and images for: MicroRNA-15a-5p acts as a tumor suppressor in histiocytosis by mediating CXCL10-ERK-LIN28a-let-7 axis
Source: Leukemia. 2021 Nov 16;36(4):1139–49. doi: 10.1038/s41375-021-01472-2 (PMC8979810; doi:10.1038/s41375-021-01472-2)

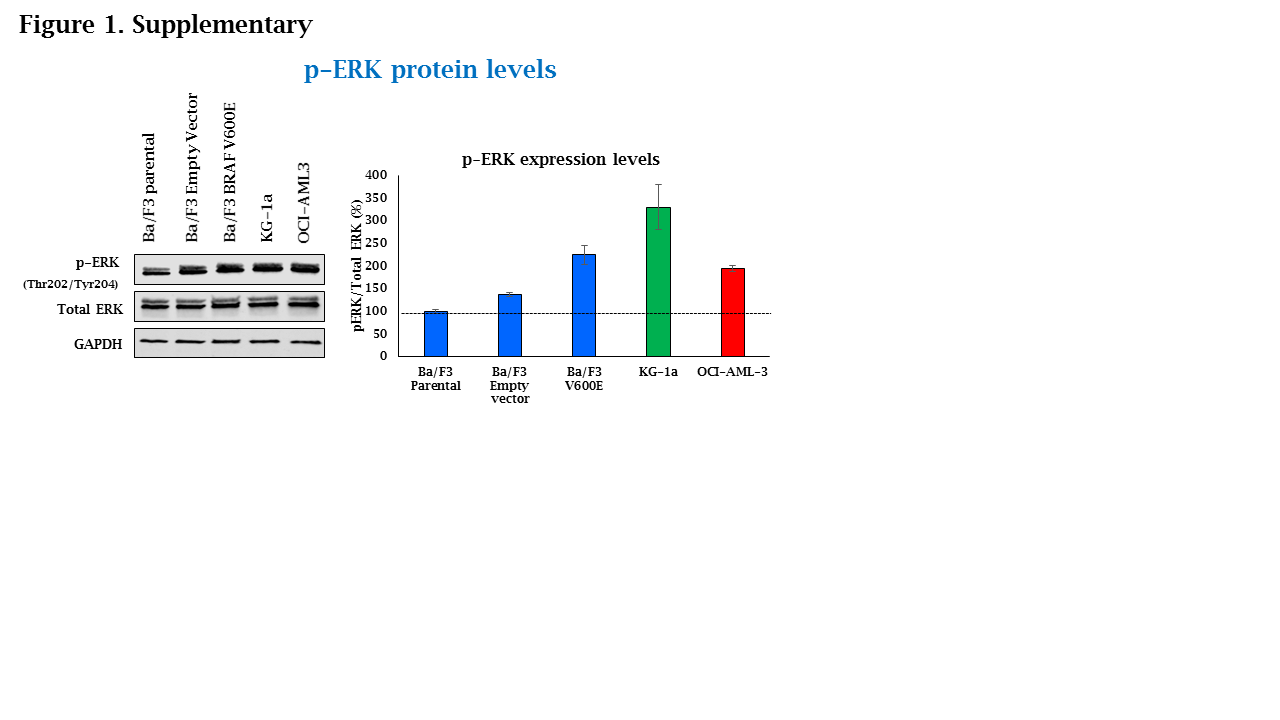

Supplement: Supplementary file 2 — Supplementary Figure 1 [file 41375_2021_1472_MOESM2_ESM.tif]
