# Supplementary material for: MicroRNA-15a-5p acts as a tumor suppressor in histiocytosis by mediating CXCL10-ERK-LIN28a-let-7 axis
Source: Leukemia. 2021 Nov 16;36(4):1139–49. doi: 10.1038/s41375-021-01472-2 (PMC8979810; doi:10.1038/s41375-021-01472-2)
Supplement: Supplementary file 3 — Supplementary figure legend [file 41375_2021_1472_MOESM3_ESM.docx]

**Supplementary figure legend**

**Supplementary Figure 1.** Phosphorylated ERK (p-ERK) protein levels in KG-1a, OCI-AML3, and Ba/F3 cells infected with an empty vector or with a *BRAF V600E* overexpressing construct. Protein samples were prepared and resolved by SDS-PAGE. p-ERK, total ERK and GAPDH (loading control) antibodies were used to detect protein levels. Ba/F3 cells infected with a *BRAF V600E* construct, KG-1a and OCI-AML3 cells showed high levels of p-ERK compared to the naïve cells and the empty vector-infected cells.
